# Tunicamycin specifically aggravates ER stress and overcomes chemoresistance in multidrug-resistant gastric cancer cells by inhibiting N-glycosylation

**DOI:** 10.1186/s13046-018-0935-8

**Published:** 2018-11-09

**Authors:** Jian Wu, Sheng Chen, Hao Liu, Zhe Zhang, Zhen Ni, Jie Chen, Zhiping Yang, Yongzhan Nie, Daiming Fan

**Affiliations:** State Key Laboratory of Cancer Biology, National Clinical Research Center for Digestive Diseases and Xijing Hospital of Digestive Diseases, Fourth Military Medical University, 127 West Changle Road, Xi’an, 710032 Shaanxi China

**Keywords:** Gastric cancer, Multidrug resistance, Tunicamycin, Glycosylation, ER stress, Autophagy

## Abstract

**Background:**

Multidrug resistance remains a major obstacle to successful treatment for patients with gastric cancer (GC). Recently, glycosylation has been demonstrated to play a vital role in the acquisition of multidrug resistance. As a potent inhibitor of glycosylation, tunicamycin (Tu) has shown marked antitumor activities in various cancers. In the present study, we attempted to determine the exact effect of Tu on the chemoresistance of GC.

**Methods:**

The cytotoxic effects of drugs on GC cells were evaluated by cell viability assays, and apoptosis was detected by flow cytometry. PCR, western blot analysis, immunofluorescence staining and canonical inhibitors were employed to identify the underlying mechanisms of the specific effects of Tu on multidrug-resistant (MDR) GC cells.

**Results:**

For the first time, we found that MDR GC cells were more sensitive to Tu-induced cell death than the parental cells and that the increased sensitivity might correlate with basal endoplasmic reticulum (ER) stress. In addition, Tu dramatically increased chemotherapy-induced apoptosis by evoking ER stress in GC cells, particularly MDR cells. Further study indicated that these effects were highly dependent on glycosylation inhibition by Tu, rather than its role as a canonical ER stress inducer. Besides, autophagy was markedly triggered by Tu, and blocking autophagy enhanced the combined effects of Tu and chemotherapy on MDR GC cells.

**Conclusions:**

Our results suggest that tumor-targeted glycosylation inhibition may be a feasible strategy to reverse chemoresistance in GC patients.

**Electronic supplementary material:**

The online version of this article (10.1186/s13046-018-0935-8) contains supplementary material, which is available to authorized users.

## Background

Gastric cancer (GC) is the second leading cause of cancer-related mortality in China and one of the most common causes of cancer-related deaths worldwide [1, 2]. Despite the substantial improvements made in the screening and treatment of GC in recent decades, it remains a devastating disease with dismal survival rates [3]. The development of multidrug resistance is a major reason for the poor prognosis of GC patients. Thus, it is imperative to identify the Achilles’ heel of multidrug resistance that could be exploited for the development of more effective therapeutics to treat GC patients.

As a major post-translational modification (PTM), glycosylation plays a vital role in the folding, stability, subcellular localization and biological functions of glycoproteins. At present, aberrant glycosylation has been widely recognized as an important hallmark of cancer and significantly correlates with the development, progression, metastasis and chemoresistance of tumors [4–12]. Our previous studies demonstrated that the dysregulated glycosylation of P-gp greatly impacted its function in the multidrug resistance of GC [13], and the aberrant glycosylation of secreted proteins might be involved in the development of multidrug resistance in GC cells [14]. Initially identified as a natural antibiotic, tunicamycin (Tu) is also a canonical compound for blocking N-linked glycosylation by inhibiting the transfer of UDP-N-acetylglucosamine (GlcNAc) to dolichol phosphate in the endoplasmic reticulum (ER) of eukaryotic cells, thus disrupting protein maturation [15–17]. Based on these, Tu has been identified as a promising anticancer therapeutic. It was reported that Tu could promote apoptosis and sensitize cancer cells to chemotherapy and radiation therapy [18–21]. Other studies showed that Tu treatment could reverse chemoresistance in several cancers, at least partially [22–24]. Wojtowicz et al. showed that Tu significantly increased the chemotherapy-induced death of drug-resistant cancer cells by regulating the function and localization of P-gp and BCRP [22].

Moreover, by inhibiting protein glycosylation, Tu could potently trigger ER stress. The ER is a dynamic network of tubules involved in the synthesis, folding and processing of over a third of the total cellular proteome. In tumor cells, the protein-processing machinery in the ER is often challenged by either intrinsic stresses, such as oncogenic activation and point mutation, or extrinsic perturbations, such as hypoxia, nutrient deprivation and acidosis. All of these would alter protein homeostasis and lead to the accumulation of misfolded proteins in the ER lumen, a state known as ER stress. Upon the initiation of ER stress, cells evoke a series of adaptive mechanisms to enhance the folding and clearance capacities, thus restoring ER proteostasis; this process is called the unfolded protein response (UPR). The UPR is mediated by three stress sensors located in the ER, namely activating transcription factor 6 (ATF6), PKR-like ER kinase (PERK) and inositol-requiring enzyme1 (IRE1). Although the UPR is initially activated as a cytoprotective response, prolonged ER stress can result in apoptosis [25–27]. Studies have shown that Tu-induced ER stress can commit cells to apoptosis [23, 24, 28]. However, there have been limited studies regarding the effects of Tu on GC.

Here, we report that Tu preferentially triggers cell death in multidrug-resistant (MDR) GC cells and significantly enhances apoptosis induced by chemotherapy through exacerbating ER stress. Moreover, we find that these specific effects are highly dependent on the inhibition of glycosylation by Tu. In addition, blocking ER stress-induced autophagy markedly increases the apoptosis of MDR GC cells under combined treatment with Tu and chemotherapy. Therefore, we believe that developing novel drugs that specifically inhibit glycosylation in tumor cells will improve the efficacy of chemotherapy for GC patients in the clinic, which deserves further research.

## Methods

### Reagents and antibodies

Adriamycin (Adr), vincristine sulfate (Vcr), hydroxychloroquine Sulfate (HCQ) and brefeldin A (BFA) were purchased from Selleck Chemicals (Houston, TX, USA). Tunicamycin (Tu, ab120296) was purchased from Abcam (Cambridge, MA, USA). Thapsigargin (Tg, #12758) was from Cell Signaling Technology (Danvers, MA, USA). Tu, Tg and BFA were dissolved in DMSO and then diluted with RPMI-1640 medium for subsequent experiments. Additionally, Adr, Vcr and HCQ were dissolved in PBS and diluted with RPMI-1640 medium for further experiments. Anti-rabbit IgG (#7074), anti-mouse IgG (#7076) and antibodies against cleaved caspase-3 (#9664), cleaved caspase-7 (#8438), cleaved PARP (#5625), β-actin (#8457), PERK (#5683), IRE1α (#3294), Bip (#3177), XBP1s (#12782), PDI (#3501), CHOP (#2895), L1CAM (#89861), and TIMP1 (#8946) were from Cell Signaling Technology. Goat anti-mouse IgG Alexa Fluor® 488 (ab150113), donkey anti-rabbit IgG Alexa Fluor® 594 (ab150076) and antibodies against p-IRE1 (ab124945), LC3B (ab192890) and P62 (ab56416) were from Abcam.

### Cell culture

The human gastric adenocarcinoma cell line SGC7901 was obtained from the Academy of Military Medical Science (Beijing, China). The MDR derivatives SGC7901/ADR (ADR) and SGC7901/VCR (VCR) were developed and maintained as previously described [14]. Other gastric cancer cell lines (BGC823, MKN45, AGS) and the human immortalized gastric epithelial cell line (GES) were from the cell bank of our lab. All cell lines were cultured in RPMI-1640 medium with 10% FBS, 100 U/ml penicillin sodium and 100 mg/ml streptomycin sulfate at 37 °C in a humidified atmosphere containing 5% CO_2_.

### Cell viability assay

Cell viability was measured with Cell Counting Kit-8 (DOJINDO, Kumamoto, Japan) according to the manufacturer’s instructions. Cells were seeded in 96-well plates at a density of 3 × 10^3^ cells/well with five replicates and incubated overnight. We then treated the cells with the appropriate drugs. After treatment, the original medium was replaced with a mixture of 10 μl CCK-8 reagent and 100 μl fresh medium. Cells were incubated for another 4 h at 37 °C. Finally, the absorbance of each well was measured by a microplate reader (Varioskan Flash, Thermo Scientific) at 450 nm. Every experiment was performed in triplicate.

### Apoptosis assay by flow cytometry

GC cells were grown in 6-well plates and treated with the appropriate chemicals for the desired period. After treatment, the cells were harvested and then resuspended in binding buffer. Afterwards, the cells were incubated with Annexin V-FITC and PI (Beyotime, China) according to the manufacturer’s instructions. Subsequently, the cells were analyzed with a flow cytometer (FACScan, BD Biosciences, USA). All experiments were performed in triplicate.

### Western blot analysis

Protein expression was determined by western blot analysis. The cells were grown in 6-well plates and then lysed with RIPA buffer (Beyotime, Shanghai, China) containing a complete® protease inhibitor cocktail (Roche, Manheim, Germany). The protein concentration was determined using a BCA kit (Thermo Scientific, USA). Protein samples (30 μg) were separated on SDS-PAGE gels and then transferred to PVDF membranes (Thermo Scientific, USA). Afterwards, the membranes were blocked in 5% nonfat milk and incubated with antibodies as described in our previous work [14]. Protein signals were detected by the ChemiDoc XRS+ imaging system (BIO-RAD, CA, USA) using the ECL reagent (Millipore, MA, USA) and quantified by Image Lab software (BIO-RAD). All experiments were performed in triplicate.

### Immunofluorescence analysis

The cell immunofluorescence assay was performed with a CST immunofluorescence application solutions kit (#12727) according to the standard protocol. After incubation with antibodies, the cells were counterstained with ProLong® Gold Antifade Reagent with DAPI (#8961). Then, the cells were visualized and photographed using an Olympus confocal laser scanning microscope FV1200.

### PCR array and data analysis

Changes in UPR-related genes were determined by the RT^2^ Profiler™ PCR Array-Human Unfolded Protein Response (PAHS-089Z, Qiagen). Cells were grown in 6-well plates and subjected to treatments for 48 h before being harvested for further analysis. Total RNA was isolated using the Qiagen RNeasy Mini Kit, and cDNA was synthesized using the Qiagen RT^2^ First Strand Kit. The subsequent procedures followed the instructions provided by Qiagen. The reactions were performed with Bio-Rad CFX96. The web-based analysis tool of Qiagen was used to interpret the results. In addition, GO analysis of the identified genes was conducted with the PANTHER classification system.

### Statistical analysis

The data are expressed as the mean ± SD. Statistical analysis was performed using one-way ANOVA in GraphPad Prism (version 7.0). Differences were considered statistically significant when P was < 0.05.

## Results

### Basal ER stress determines the higher sensitivity of MDR cells to Tu

**Fig. 1:**
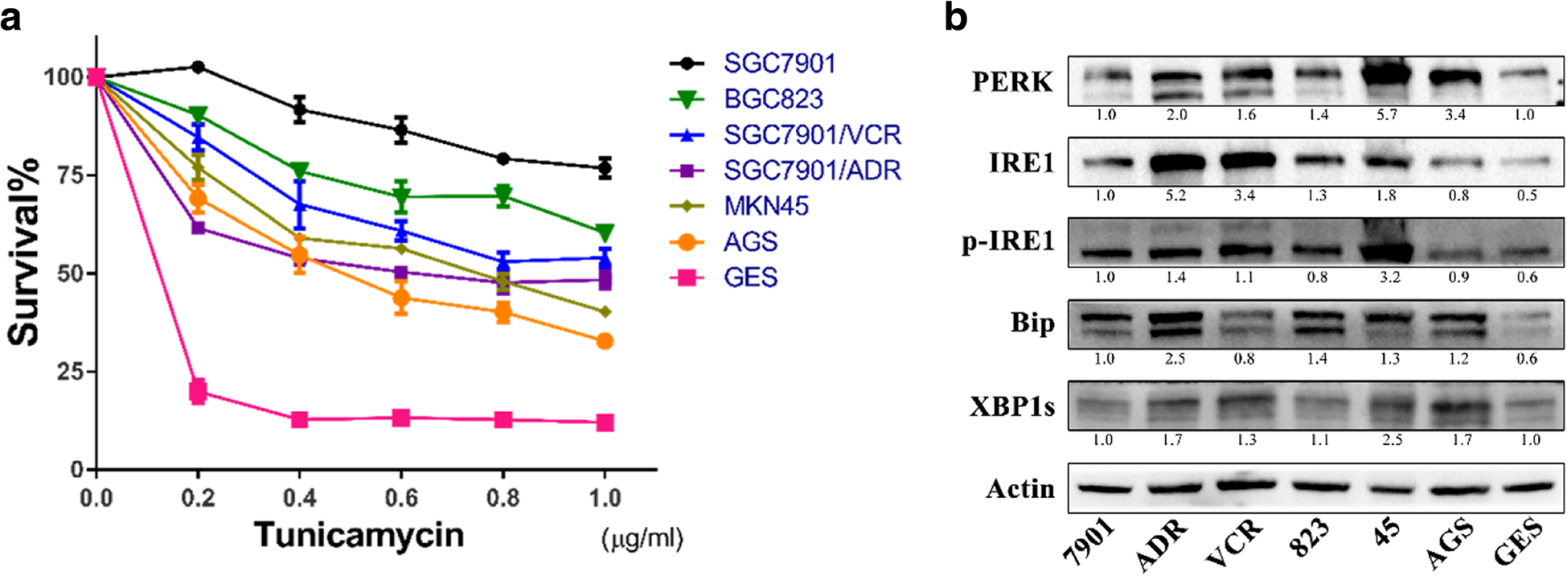
Tunicamycin exerts strong cytotoxic effects on MDR GC cells, which correlates with basal ER stress. **a** Cell survival after Tu treatment for 48 h assayed by CCK-8. Tu, tunicamycin. The values in the untreated group are identified as the baseline, namely 100% survival. **b** Expressions of ER stress-associated proteins in cells without treatment. β-actin served as the loading control. All proteins were normalized to β-actin. ER, endoplasmic reticulum; 7901, SGC7901; ADR, SGC7901/ADR; VCR, SGC7901/VCR; 823, BGC823; 45, MKN45; GES, human immortalized gastric epithelial cell line

To explore the effects of Tu on GC cells, we identified the dose-response curves of GES and 6 gastric cancer cell lines, including two derivative MDR cell lines. In general, the cytotoxicity assay indicated that GES was more sensitive to Tu than any other cell line, demonstrating the more toxic effects of Tu on normal tissues. Moreover, as for the MDR cell models, MDR cells (SGC7901/ADR and SGC7901/VCR) showed higher sensitivity to Tu than the parental cells (SGC7901) (Fig. 1a).

Given that Tu can activate ER stress and that excess ER stress leads cells to death, we determined the expressions of ER stress-associated proteins in cells without treatment by western blot (WB) analysis. The results showed that PERK, p-IRE1, IRE1 and XBP1s were all upregulated in MDR cells compared to those in the parental cells, except for Bip. Bip was slightly downregulated in SGC7901/VCR compared to SGC7901. When the expressions of all the proteins were considered comprehensively, we believed that compared to the parental cells, MDR GC cells displayed higher levels of basal ER stress (Fig. 1b), which could be the determinant factor driving their greater susceptibility to Tu. That deserves further exploration.

### Tu preferentially induces MDR cells to death in a time/dose-dependent manner by aggravating ER stress

**Fig. 2:**
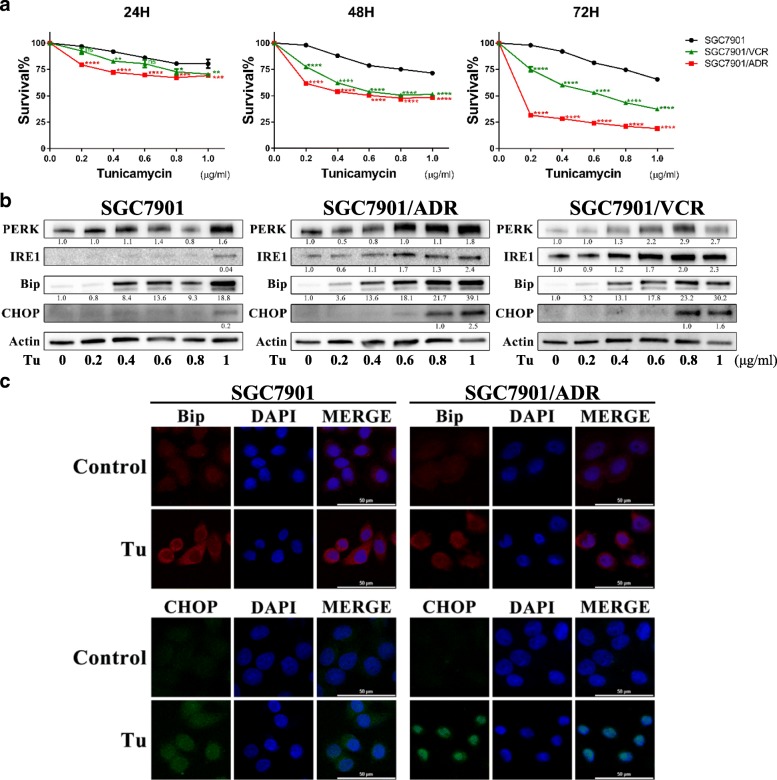
Tunicamycin preferentially leads MDR GC cells to death in a time/dose-dependent manner via inducing ER stress. **a** Survival of GC cells after Tu treatment for 24/48/72 h assayed by CCK-8. ns, non-significant; *P < 0.05, **P < 0.01, ***P < 0.001, ****P < 0.0001 (green, VCR versus 7901; red, ADR versus 7901). **b** Expressions of ER stress-associated proteins in GC cells after Tu treatment for 48 h. All proteins were normalized to β-actin. **c** Expressions of Bip and CHOP in SGC7901 and SGC7901/ADR detected by immunofluorescence (IF) after Tu (0.8 μg/ml) treatment for 48 h. (400 ×; scale bar, 50 μm)

We treated the two MDR cell lines (SGC7901/ADR and SGC7901/VCR) and their parental cell line SGC7901 with Tu (0–1 μg/ml) for 24, 48 and 72 h to obtain more detailed information about their responses to Tu. Generally, we found that Tu preferentially reduced the viability of MDR cells in a time/dose-dependent manner (Fig. 2a). Furthermore, after exposure to Tu for 48 h, MDR cells exhibited more significant enhancements of ER stress-associated proteins (PERK, IRE1, Bip, CHOP) with increasing doses, as demonstrated by WB analysis (Fig. 2b) and immunocytochemical staining (Fig. 2c). Based on the above results, we believed that Tu could specifically exacerbate basal ER stress in MDR cells, which made them more vulnerable to Tu treatment.

### Tu potently increases drug-induced apoptosis in MDR cells via further enhancing ER stress

**Fig. 3:**
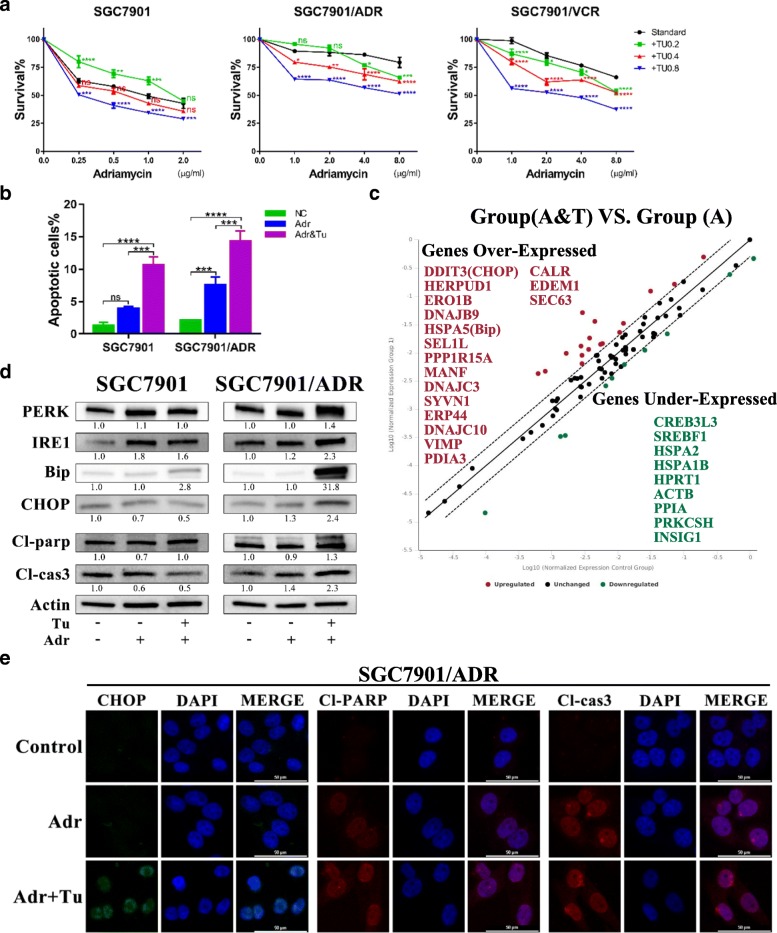
Tunicamycin increases chemotherapy-induced cell death by aggravating ER stress and enhancing apoptosis. **a** Survival of GC cells measured by CCK-8 after treatment with monotherapy (Adr) or dual therapy (Adr and Tu) for 48 h. Adr, adriamycin; the concentrations of Tu were 0/0.2/0.4/0.8 μg/ml. ns, non-significant; *P < 0.05, **P < 0.01, ***P < 0.001, ****P < 0.0001 (green, +Tu 0.2 versus standard; red, +Tu 0.4 versus standard; blue, +Tu 0.8 versus standard). **b** Apoptotic cells detected by flow cytometry. Cells were subjected to monotherapy (Adr, 0.25/8 μg/ml for SGC7901 and SGC7901/ADR, respectively) or dual therapy (Adr, same as the former; Tu, 0.8 μg/ml for both) for 48 h before the apoptosis assay. ns, non-significant; ***P < 0.001, ****P < 0.0001. The corresponding FCM graphs were shown in Additional file 7: Figure S6. **c** Changes in UPR-related genes assayed by PCR array in SGC7901/ADR (dual therapy group versus monotherapy group). Group (A & T), dual therapy with Adr (8 μg/ml) and Tu (0.8 μg/ml) for 48 h; Group (A), monotherapy with Adr (8 μg/ml) for 48 h, the control group. The colored dots represent over-expressed or under-expressed genes; the black dots represent unchanged genes. *P* < 0.05. **d** Expressions of proteins involved in the UPR and apoptosis signaling determined by WB in SGC7901 and SGC7901/ADR. Cells were subjected to the same treatments as above (3b) before protein extraction. All proteins were normalized to β-actin. **e** Expression levels of CHOP, Cl-PARP and Cl-caspase 3 in SGC7901/ADR detected by IF after treatment with monotherapy or dual therapy for 48 h. The concentrations of drugs were the same as those in 3c. (400 ×; scale bar, 50 μm)

Because MDR GC cells were more susceptible to Tu, we next explored whether Tu could overcome chemoresistance in GC cells. Cell viability assays indicated that cotreatment of Tu (0.2/0.4/0.8 μg/ml) with Adr could significantly reduce cell survival in MDR cells at any dose of Tu, whereas only the large concentration of Tu (0.8 μg/ml) could markedly enhance cell death in the parental cell line (Fig. 3a). To further identify the interaction between Tu and Adr, we calculated the Combination index (CI) values (Additional file 1: Table S1) for GC cells using CompuSyn software. Disappointingly, despite the fact that combined therapy with Tu and Adr could induce more cell death than monotherapy with Adr, the combination did not indicate synergistic effects for MDR cells. Despite this, dual therapy with Tu and Adr triggered significantly more apoptosis of GC cells than monotherapy with Adr (Fig. 3b). Taken together, these results showed that Tu could decrease the chemoresistance of MDR cells to some extent.

To elucidate the underlying mechanism, we performed the PCR array for SGC7901 and SGC7901/ADR after treatment with Adr alone or Adr and Tu. The results showed that in SGC7901/ADR, the differentially expressed genes between the CT (combined therapy with Tu and Adr) group and the Adr group were mainly involved in the UPR and apoptosis signaling pathways (Fig. 3c), which was further evidenced by GO analysis of these identified genes (Additional file 2: Figure S1). However, we found few changes in such genes in SGC7901 between the CT group and Adr group (Additional file 3: Figure S2).

Moreover, at the protein level, we carried out WB and immunofluorescence (IF) experiments to explore the changes in UPR- and apoptosis-related proteins in SGC7901 and SGC7901/ADR after monotherapy (Adr) or CT (Adr and Tu). Our results indicated that Bip (GRP78), an important modulator of ER stress, was significantly increased in the CT group of SGC7901/ADR compared to the Adr group. We also identified the notable up-regulation of CHOP (C/EBP homologous protein), a key player in UPR-mediated apoptotic pathway, in the CT group of SGC7901/ADR. In addition, the effects of CT in SGC7901/ADR were further evidenced by increases in cleaved caspases and PARP (Fig. 3d). Furthermore, in SGC7901/ADR, IF assays of CHOP, Cl-caspase3 and Cl-PARP also demonstrated marked enhancements in the CT group compared to the Adr group (Fig. 3e). However, the increases in these proteins were not as significant in the CT group of SGC7901 (Fig. 3d and Additional file 4: Figure S3). Generally, our results demonstrated that CT could induce more apoptosis by aggravating ER stress in MDR GC cells, thus reversing chemoresistance.

### The specific impacts of Tu on MDR cells strongly depend on its inhibition of N-glycosylation

As already reported, Tu potently induces ER stress by inhibiting the N-linked glycosylation of glycoproteins. However, thapsigargin (Tg) functions as another ER stress inducer by disrupting Ca^2+^ homeostasis in the ER. Hence, to determine whether the specific effects of Tu on MDR GC cells were mainly due to its inhibition of N-glycosylation or its role as an ER stress inducer, we attempted to mimic its particular influences on MDR cells using Tg.

**Fig. 4:**
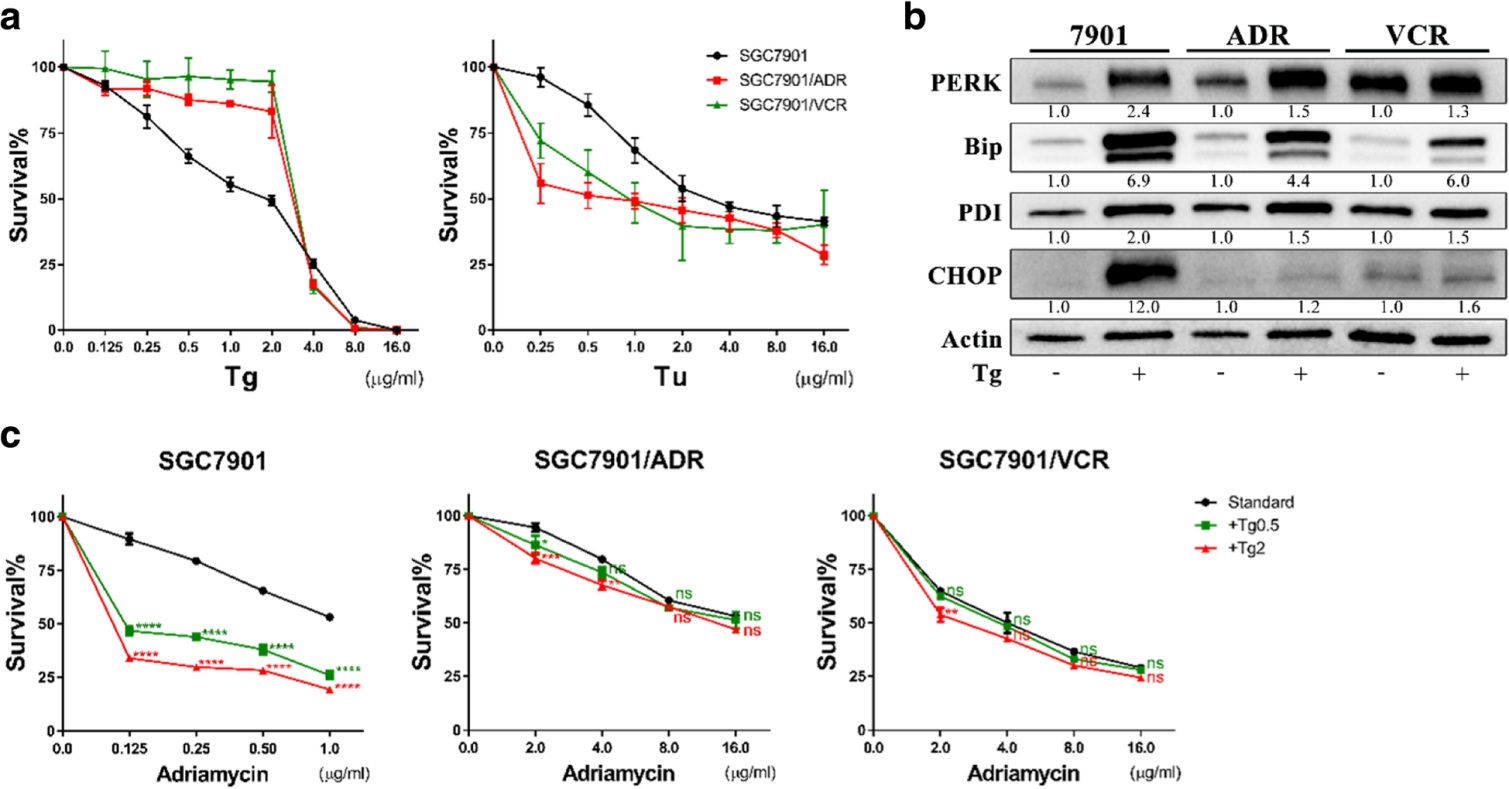
Thapsigargin could not mimic the effects of tunicamycin on MDR GC cells. **a** Concentration-survival curves of GC cells treated with Tg or Tu (wide dose range) for 48 h. Tg, thapsigargin. **b** Expressions of UPR-related proteins in GC cells after Tg treatment (2 μg/ml) for 48 h determined by WB. All proteins were normalized to β-actin. **c** The effects of Tg on the chemosensitivity of GC cells after treatment for 48 h. ns, non-significant; *P < 0.05, **P < 0.01, ***P < 0.001, ****P < 0.0001 (green/red, +Tg 0.5/2 versus standard, respectively)

First, we compared the responses of SGC7901, SGC7901/ADR and SGC7901/VCR to Tg and Tu, respectively, across large dosage ranges (0.125–16 μg/ml for Tg; 0.25–16 μg/ml for Tu). The results of CCK-8 assays showed that the two MDR cell lines SGC7901/ADR and SGC7901/VCR were less sensitive to Tg compared to the parental cell line, which was opposite to the effects of Tu on these 3 cell lines (Fig. 4a).

In addition, to verify its function of inducing ER stress, we incubated the 3 cell lines with Tg (2 μg/ml) for 48 h and subsequently determined the expressions of UPR-related proteins by WB. The results demonstrated that Tg could markedly activate ER stress, especially in SGC7901 (Fig. 4b).

Furthermore, we treated the 3 cell lines with dual therapy of Adr and Tg (0.5/2 μg/ml) to explore whether Tg could potentiate the chemosensitivity of MDR cells to Adr. However, the cell viability assays indicated that Tg notably increased chemotherapy-induced cell death of SGC7901 at any dose but had little influence on the survival of MDR cells undergoing Adr treatment (Fig. 4c).

In addition, we employed another glycosylation inhibitor to further demonstrate the significance of glycosylation inhibition in the specific effects of Tu on MDR cells. First, we further illustrated the inhibitory effects of Tu on N-glycosylation by investigating example glycoproteins (Additional file 5: Figure S4a). L1CAM and TIMP1 are two identified glycoproteins modified with N-glycans, as recorded in the Uniprot database. Our previous study discovered that the N-glycosylation status of L1CAM and TIMP1 dramatically changed after the acquisition of chemoresistance in GC cells [14]. Thus, we explored whether Tu could inhibit the glycosylation of these two glycoproteins in GC cells. The WB results indicated that the expressions of L1CAM and TIMP1 decreased after Tu treatment (0.8 μg/ml) for 48 h in GC cells, particularly MDR cells (Additional file 5: Figure S4a). In addition, as indicated by Uniprot and our work [14], L1CAM possesses more glycosites and is modified with more N-glycans. As a result, the expression of L1CAM is more vulnerable to glycosylation inhibition. Accordingly, the WB bands of L1CAM showed an obvious downward shift in MDR GC cells after Tu treatment (Additional file 5: Figure S4a). These results demonstrated that Tu could potently inhibit the N-glycosylation of glycoproteins in GC cells. Brefeldin A (BFA) can block protein transport from the ER to the Golgi apparatus and thus inhibit the glycosylation process. Therefore, we explored the effects of BFA on GC cells. The results demonstrated that MDR cells were more vulnerable to BFA treatment (Additional file 5: Figure S4b), and BFA could inhibit the glycosylation of L1CAM and activate ER stress in GC cells (Additional file 5: Figure S4c). More importantly, BFA significantly increased the chemosensitivity of GC cells to Adr (Additional file 5: Figure S4d). In summary, our results indicated that BFA could mimic the effects of Tu on MDR cells to some extent.

In conclusion, our results demonstrated that compared to Tg, BFA could imitate the effects of Tu on MDR GC cells, which indicated that the inhibition of N-glycosylation by Tu determined its specific impacts on MDR cells rather than its role as an ER stress inducer.

### Blocking autophagy enhances the inhibitory effects of Tu and Adr on GC cells

At present, it is widely accepted that ER stress is a potent inducer of autophagy [29–31]. As Tu could significantly activate ER stress in GC cells (as evidenced above), we sought to determine whether Tu-induced ER stress triggered autophagy and the exact role of autophagy in the effects of Tu on GC cells.

**Fig. 5:**
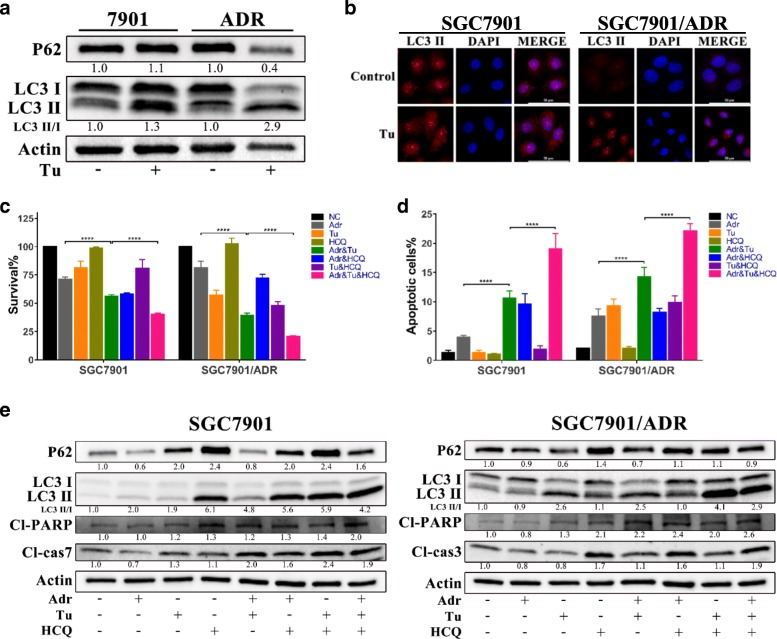
Tu-induced autophagy antagonizes the chemosensitizing effects of Tu on MDR GC cells. **a** Expressions of LC3 II and P62 in GC cells after Tu treatment (0.8 μg/ml) for 48 h, as detected by WB. All proteins were normalized to β-actin. **b** Expression of LC3 II in GC cells after Tu treatment (0.8 μg/ml) for 48 h, as detected by IF. (400 ×; scale bar, 50 μm.) **c** The effects of autophagy blockade on the survival of GC cells assayed by CCK-8. HCQ, hydroxychloroquine; Tu, 0.8 μg/ml; HCQ, 25 μM; Adr, 0.25 μg/ml for 7901, 8 μg/ml for ADR. Cells were subjected to treatments for 48 h before the CCK-8 assay. ****P < 0.0001. **d** The effects of autophagy blockade on the apoptosis of GC cells, as determined by flow cytometry. The treatments were the same as those in 5c. ****P < 0.0001. **e** Expression levels of proteins involved in autophagy and apoptosis signaling in GC cells after treatments for 48 h. The treatments were the same as those in 5c. All proteins were normalized to β-actin

We incubated SGC7901 and SGC7901/ADR with Tu (0.8 μg/ml) for 48 h and then subjected their cell lysates to WB for detection of LC3II/LC3I and P62, which are common markers of autophagy. The results indicated a marked increase in the ratio of LC3II/LC3I and a corresponding decrease in P62 in SGC7901/ADR after Tu treatment but no obvious changes in SGC7901 (Fig. 5a). In addition, the fluorescence of LC3II was also markedly enhanced in SGC7901/ADR after 48 h treatment of Tu, as shown in Fig. 5b.

We used hydroxychloroquine (HCQ), a lysosome inhibitor, to block autophagic flux, and then identified whether autophagy inhibition impacted the effects of Tu on GC cells. Firstly, we chose 25 μM HCQ for further experiments because HCQ effectively inhibited Tu-induced autophagy and exerted little effect on the viability of GC cells at 25 μM (Additional file 6: Figure S5). Fig. 5c showed that HCQ significantly decreased the cell viability of GC cells when combined with the dual therapy of Adr and Tu. Moreover, we discovered that HCQ markedly increased the apoptosis induced by dual therapy of Adr and Tu (Fig. 5d).

Furthermore, WB analysis showed that after the addition of HCQ, the expressions of cleaved caspases and PARP increased compared to those in the CT (Adr and Tu) group in GC cells. Besides, we also found a substantial increase in the LC3II/LC3I ratio in GC cells with triple therapy (Adr, Tu and HCQ) (Fig. 5e), which might have resulted from the simultaneous autophagy induction by drugs and autophagic flux blockade by HCQ.

After comprehensive analysis, we found that Tu could significantly trigger autophagy in MDR cells and that HCQ enhanced the combined effects of Adr and Tu by inhibiting Tu-induced autophagy. However, in SGC7901, Adr activated autophagy, and HCQ promoted the inhibitory effects of Adr and Tu by blocking Adr-induced autophagy.

In summary, Tu-induced autophagy did not contribute to the effects of Tu on MDR GC cells but rather antagonized its chemosensitizing impacts. Autophagy inhibition could further potentiate the cell death of GC cells induced by CT with Adr and Tu.

## Discussion

ER stress closely correlates with many hallmarks of cancer, such as angiogenesis, invasion, proliferation and survival, in many types of tumors [25–27, 32]. In addition, a large number of studies have demonstrated the tight relationship between ER stress and chemoresistance in various cancers [33–40]. For example, as one of the three major effectors of UPR, ATF6α activation plays a vital role in conferring imatinib resistance on leukemia cells, which is regulated by protein disulfide isomerase A5 (PDIA5). In addition, inhibition of the PDIA5/ATF6α activation loop is able to restore chemosensitivity [37]. Our results revealed that MDR GC cells displayed a high level of basal ER stress, as evidenced by the increases in UPR-associated proteins, implying a correlation between ER stress and chemoresistance in GC. Although ER stress is initially activated as a cytoprotective mechanism, excess or prolonged ER stress can result in apoptosis [25, 26, 32]. Based on this, it seemed that the basal ER stress made MDR cells more vulnerable to Tu-induced cell death compared to the parental cells. In the present study, we found that Tu could preferentially direct MDR cells to death in a time/dose-dependent manner by aggravating ER stress.

Recent studies have demonstrated that Tu could enhance the antitumor efficacy of both chemotherapy and molecular targeted therapy [21, 41]. For example, Tu sensitized hepatocellular carcinoma to cisplatin-induced apoptosis and reversed drug resistance by regulating the DPAGT1/Akt/ABCG2 pathway [21]. Consistent with these findings, our results demonstrated that cotreatment with Tu and Adr dramatically decreased the viability of GC cells, especially MDR cells, by triggering ER stress-associated apoptosis. Thus, our findings indicated that Tu could overcome chemoresistance to some extent by enhancing apoptosis in MDR GC cells.

To define the molecular mechanisms underlying the specific effects of Tu on MDR GC cells, we employed Tg, another widely used ER stress inducer, to imitate the effects of Tu on GC cells. Tu strongly interferes with the N-linked glycosylation of proteins in the ER and thus potently induces ER stress. Tg triggers ER stress by blocking the sarco/endoplasmic reticulum Ca^2+^ (SERCA)-adenosine triphosphatase (ATPase) and disrupting Ca^2+^ homeostasis [42]. Our findings demonstrated that although Tg could also markedly induce ER stress in GC cells, its effects were opposite to those of Tu. In contrast to Tu, Tg was more cytotoxic to the parental cells compared to MDR cells and specifically sensitized the parental cells to chemotherapy-induced cell death. Considering that Tu and Tg are both canonical ER stress inducers, their entirely different mechanisms of action may result in their contrasting effects on GC cells. Most likely because Tg cannot inhibit glycosylation, it exerted little effect on the MDR-associated glycoproteins, which subsequently led to the reduced effects of Tg on MDR cells. Moreover, we also employed BFA to further support these conclusions. Glycosylation occurs sequentially in the ER and Golgi apparatus [43, 44]. BFA can block protein export from the ER to the Golgi apparatus by inhibiting the formation of vesicles, resulting in incomplete glycosylation [45–47]. Our results indicated that BFA could mimic the effects of Tu on MDR cells to some extent. In addition, aberrant glycosylation of proteins activates ER stress and significantly contributes to the development of chemoresistance [4, 8, 11, 14, 48–51]. Thus, we believe that dysregulated glycosylation leads to basal ER stress in MDR GC cells and that Tu further exacerbates ER stress by inhibiting glycosylation, which may account for the specific effects of Tu on MDR GC cells.

Moreover, recent studies have demonstrated that ER stress can effectively trigger autophagy [29–31, 33, 35, 52]. The three branches of the UPR (PERK, IRE1, ATF6) all have marked significance in the induction of autophagy [30, 31, 52–54]. Autophagy (macroautophagy) is an evolutionarily conserved cellular catabolic process involving the formation of double-membrane vesicles named autophagosomes that deliver cellular proteins and organelles to lysosomes, wherein the cargos are degraded and recycled [55, 56]. Mounting evidence has demonstrated that autophagy has both cytoprotective and cytotoxic functions, which are context-dependent [30, 31, 57, 58]. Notably, it was reported that Tu-induced ER stress could activate autophagy in cancer cells through the IRE1/JNK signaling pathway [59, 60], and inhibiting autophagy by genetic or pharmacological means significantly enhanced the cell death induced by Tu. In contrast, blocking autophagy via bafilomycin A1 or 3-MA markedly increased the survival of mouse embryonic fibroblasts under ER stress [29]. In our study, we also discovered the upregulation of autophagy in MDR GC cells after Tu treatment. To identify the exact role of autophagy in Tu-induced cell death, we subjected GC cells to the common autophagy inhibitor HCQ, which impedes the fusion of autophagosomes with lysosomes by deacidifying lysosomes [61]. We found that autophagy blockade could enhance the inhibitory effects of Adr and Tu on GC cells, though Adr, instead of Tu, induced autophagy in the parental cells. In summary, our results indicated that autophagy played a cytoprotective role against the specific effects of Tu on MDR GC cells.

However, as Tu displayed higher toxicity in normal tissues, we did not perform in vivo studies to further corroborate the combined effects in mouse models. Next, we will collaborate with pharmacologists to develop tumor-targeted drug delivery systems for delivering Tu and other drugs to further validate these results.

## Conclusions

**Fig. 6:**
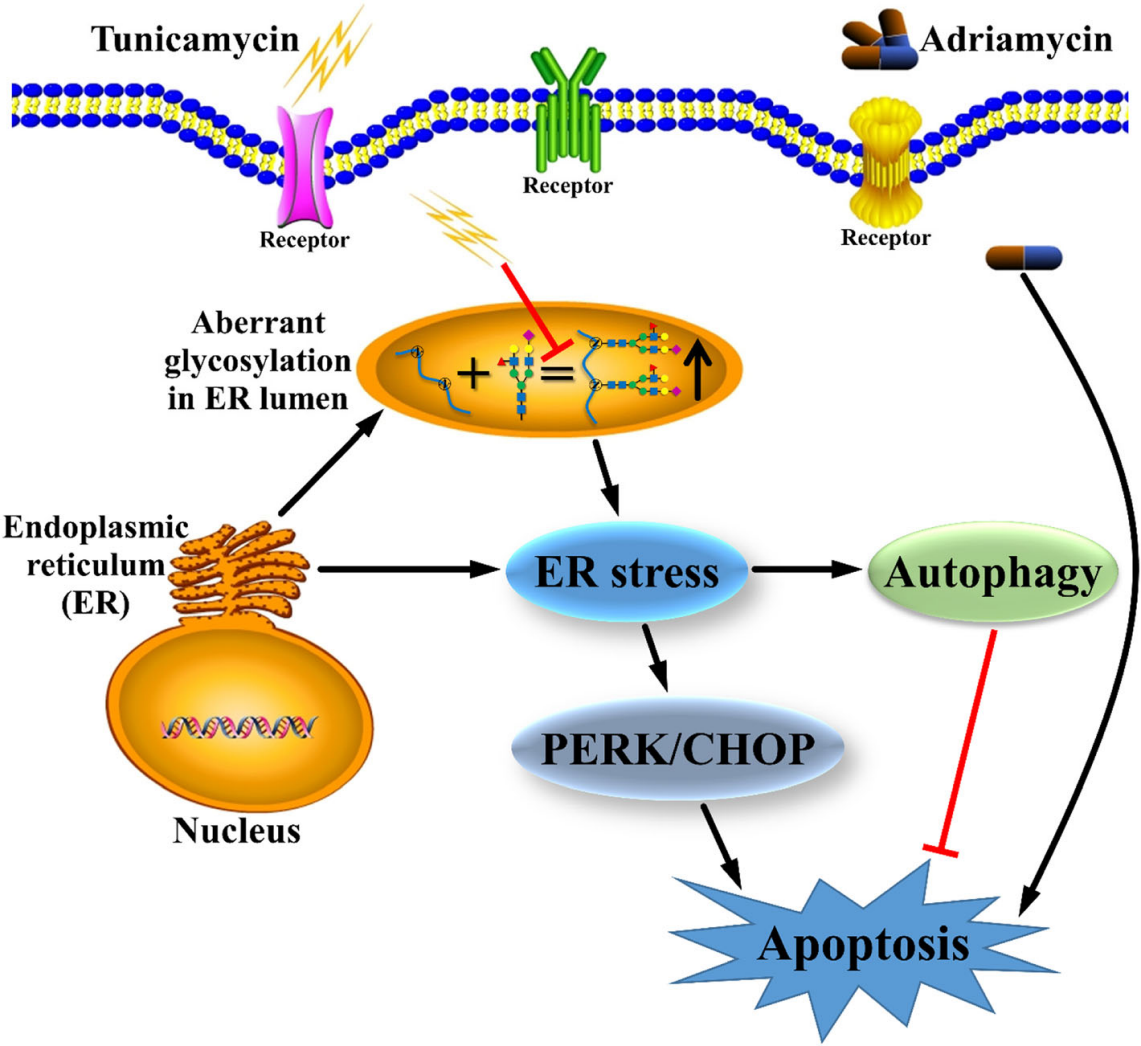
The schematic diagram illustrates the hypothetical mechanism of action of Tu in MDR GC cells. As previously reported, aberrant glycosylation contributes to chemoresistance. For example, some types of glycosylation are upregulated in MDR cells [13, 14]. Moreover, dysregulated glycosylation leads to basal ER stress in MDR GC cells. Tu further exacerbates ER stress by inhibiting N-glycosylation, and excess ER stress subsequently activates apoptosis signaling, which decreases the chemoresistance of MDR cells to some extent. However, autophagy triggered by ER stress partially dampens the apoptosis-inducing effects of Tu

In summary, we identified for the first time that Tu could preferentially commit MDR GC cells to apoptosis by inhibiting glycosylation and subsequently exacerbating ER stress (Fig. 6), thus overcoming chemoresistance to some extent. We also revealed that blocking autophagy could markedly enhance the cytotoxicity of Tu and Adr for GC cells. Our study reconfirms the importance of aberrant glycosylation in the multidrug resistance of GC and provides a new insight into the development of novel therapeutic strategies for overcoming chemoresistance in GC.

## Additional files

Additional file 1: **Table S1**. CI values for SGC7901, SGC7901/ADR and SGC7901/VCR. CI was calculated by CompuSyn, which is based on the model created by Chou and Talalay. CI < 1, CI = 1 and CI > 1 represent synergism, additive effect and antagonism, respectively. (XLSX 10 kb)

Additional file 2: **Figure S1**. GO analysis of genes significantly changed in SGC7901/ADR after treatment for 48 h (dual therapy group versus monotherapy group). (a/b) GO analysis of upregulated and downregulated genes, respectively. Dual therapy group, Adr (8 μg/ml) and Tu (0.8 μg/ml); monotherapy group, Adr (8 μg/ml), and the control group. (PPTX 301 kb)

Additional file 3: **Figure S2**. Changes in UPR-related genes in SGC7901 after treatment for 48 h (dual therapy group versus monotherapy group). Group (A & T), dual therapy with Adr (0.25 μg/ml) and Tu (0.8 μg/ml); Group (A), monotherapy with Adr (0.25 μg/ml), and the control group. The colored dots represent over-expressed or under-expressed genes; the black dots represent unchanged genes. *P* < 0.05. (PPTX 80 kb)

Additional file 4: **Figure S3**. Expression levels of CHOP, Cl-PARP and Cl-caspase 3 in SGC7901 detected by IF after treatment with monotherapy or dual therapy for 48 h. The concentrations of drugs were the same as those in Additional file 3: Figure S2. (400 ×; scale bar, 50 μm.) (PPTX 556 kb)

Additional file 5: **Figure S4**. Brefeldin A (BFA) can mimic the effects of Tu on MDR GC cells. **a** The effects of Tu on glycoproteins-L1CAM and TIMP1. GC cells were treated with Tu (0.8 μg/ml) for 48 h before harvest. All proteins were normalized to β-actin. **b** Concentration-survival curves of GC cells treated with BFA for 48 h. ns, non-significant; ****P < 0.0001 (green/red, VCR/ADR versus 7901, respectively). **c** The effects of BFA on L1CAM and UPR-related proteins in GC cells after treatment (0.02 μg/ml) for 48 h as determined by WB. All proteins were normalized to β-actin. **d** The effects of BFA on the chemosensitivity of GC cells. BFA, 0.02 μg/ml. Cells were subjected to treatments for 48 h. ****P < 0.0001. (PPTX 315 kb)

Additional file 6: **Figure S5**. HCQ (25 μM) effectively blocks Tu-induced autophagy and hardly affects the viability of GC cells. **a** Concentration-survival curves of GC cells treated with HCQ for 48 h. **b** The effects of HCQ on autophagy-related proteins in SGC7901/ADR. Cells were treated with Tu (0.8 μg/ml) or Tu and HCQ for 48 h before harvest. All proteins were normalized to β-actin. (PPTX 144 kb)

Additional file 7: **Figure S6**. Representative FCM graphs of SGC7901 (a) and SGC7901/ADR (b) corresponding to the data in Fig. 5d. The treatments were the same as those in Fig. 5d. (PPTX 368 kb)

## Abbreviations

Adr: adriamycin
BFA: brefeldin A
ER: endoplasmic reticulum
GC: gastric cancer
HCQ: hydroxychloroquine
MDR: multi-drug resistant
Tg: thapsigargin
Tu: tunicamycin
UPR: unfolded protein response
