# Supplementary figures and images for: Tunicamycin specifically aggravates ER stress and overcomes chemoresistance in multidrug-resistant gastric cancer cells by inhibiting N-glycosylation
Source: J Exp Clin Cancer Res. 2018 Nov 9;37:272. doi: 10.1186/s13046-018-0935-8 (PMC6230241; doi:10.1186/s13046-018-0935-8)

## Slide 1
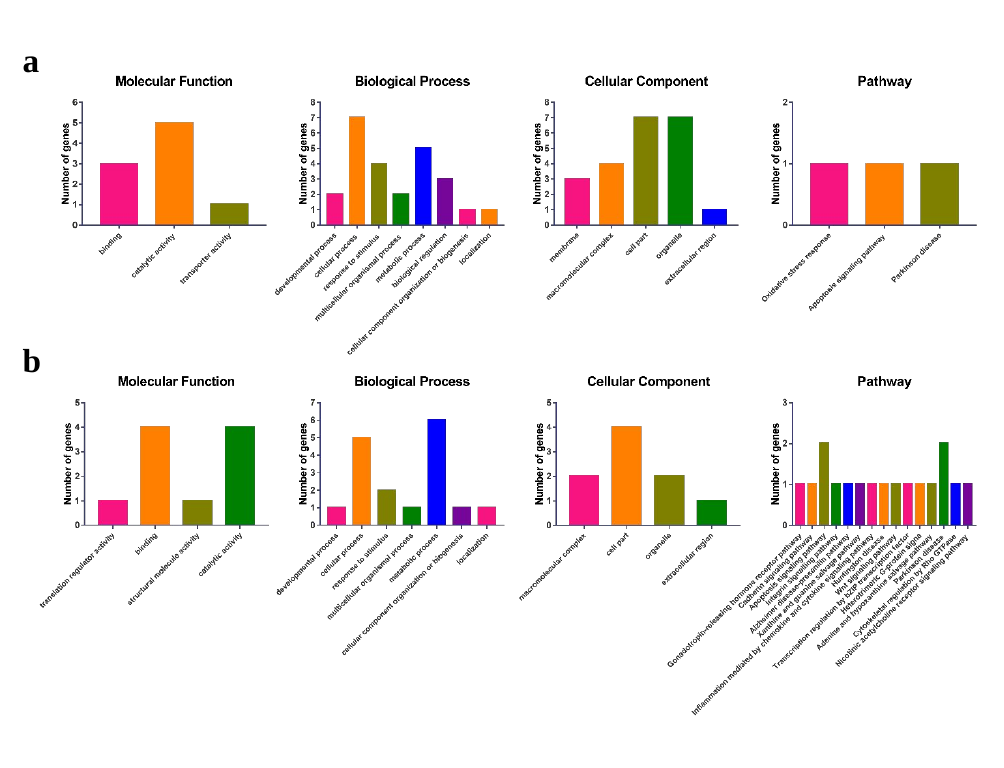

a
b

Supplement: Supplementary file 2 — Figure S1. GO analysis of genes significantly changed in SGC7901/ADR after treatment for 48 h (dual therapy group versus monotherapy group). (a/b) GO analysis of upregulated and downregulated genes, respectively. Dual therapy group, Adr (8 μg/ml) and Tu (0.8 μg/ml); monotherapy group, Adr (8 μg/ml), and the control group. (PPTX 301 kb) [file 13046_2018_935_MOESM2_ESM.pptx]

## Slide 1
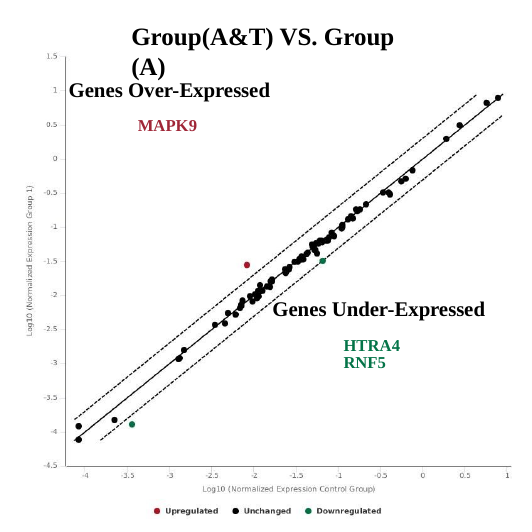

Group(A&T) VS. Group (A)
Genes Over-Expressed
MAPK9
Genes Under-Expressed
HTRA4
RNF5

Supplement: Supplementary file 3 — Figure S2. Changes in UPR-related genes in SGC7901 after treatment for 48 h (dual therapy group versus monotherapy group). Group (A & T), dual therapy with Adr (0.25 μg/ml) and Tu (0.8 μg/ml); Group (A), monotherapy with Adr (0.25 μg/ml), and the control group. The colored dots represent over-expressed or under-expressed genes; the black dots represent unchanged genes. P < 0.05. (PPTX 80 kb) [file 13046_2018_935_MOESM3_ESM.pptx]

## Slide 1
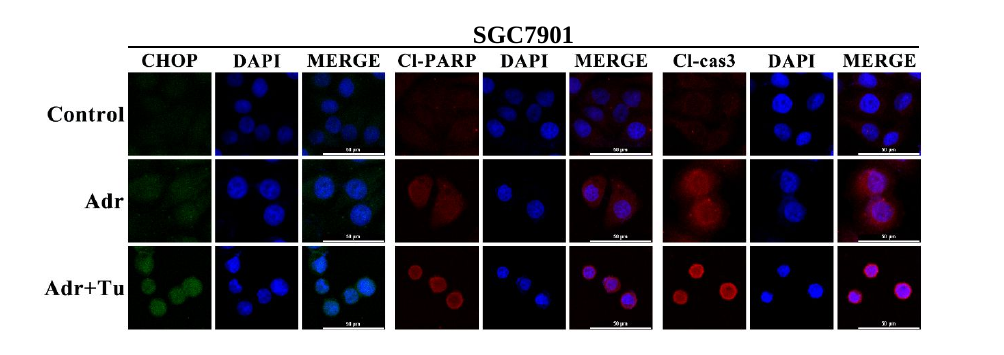

SGC7901

Supplement: Supplementary file 4 — Figure S3. Expression levels of CHOP, Cl-PARP and Cl-caspase 3 in SGC7901 detected by IF after treatment with monotherapy or dual therapy for 48 h. The concentrations of drugs were the same as those in Additional file 3: Figure S2. (400 ×; scale bar, 50 μm.) (PPTX 556 kb) [file 13046_2018_935_MOESM4_ESM.pptx]

## Slide 1
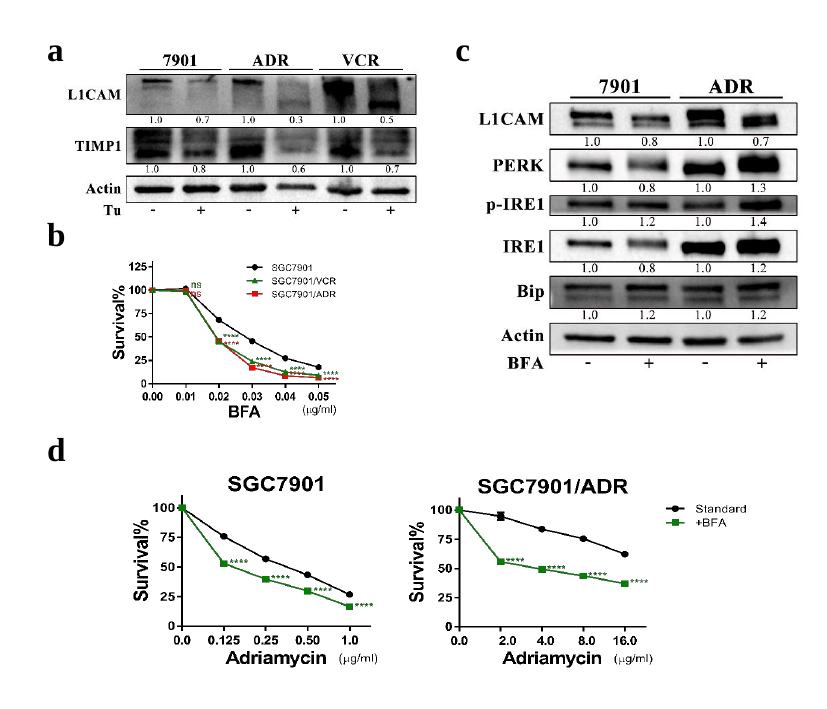

a
c
b
d

Supplement: Supplementary file 5 — Figure S4. Brefeldin A (BFA) can mimic the effects of Tu on MDR GC cells. a The effects of Tu on glycoproteins-L1CAM and TIMP1. GC cells were treated with Tu (0.8 μg/ml) for 48 h before harvest. All proteins were normalized to β-actin. b Concentration-survival curves of GC cells treated with BFA for 48 h. ns, non-significant; ****P < 0.0001 (green/red, VCR/ADR versus 7901, respectively). c The effects of BFA on L1CAM and UPR-related proteins in GC cells after treatment (0.02 μg/ml) for 48 h as determined by WB. All proteins were normalized to β-actin. d The effects of BFA on the chemosensitivity of GC cells. BFA, 0.02 μg/ml. Cells were subjected to treatments for 48 h. ****P < 0.0001. (PPTX 315 kb) [file 13046_2018_935_MOESM5_ESM.pptx]

## Slide 1
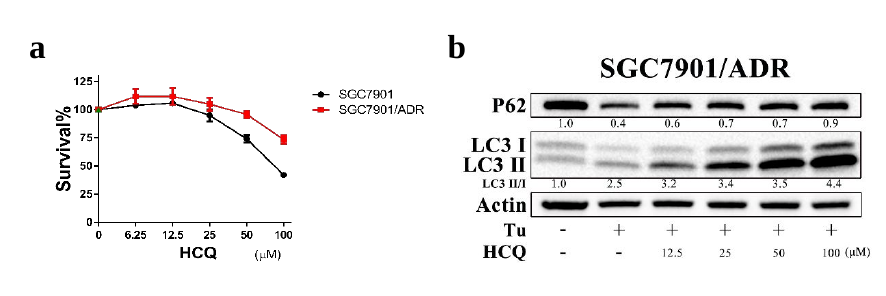

a
b

Supplement: Supplementary file 6 — Figure S5. HCQ (25 μM) effectively blocks Tu-induced autophagy and hardly affects the viability of GC cells. a Concentration-survival curves of GC cells treated with HCQ for 48 h. b The effects of HCQ on autophagy-related proteins in SGC7901/ADR. Cells were treated with Tu (0.8 μg/ml) or Tu and HCQ for 48 h before harvest. All proteins were normalized to β-actin. (PPTX 144 kb) [file 13046_2018_935_MOESM6_ESM.pptx]
