# Supplementary material for: Tunicamycin specifically aggravates ER stress and overcomes chemoresistance in multidrug-resistant gastric cancer cells by inhibiting N-glycosylation
Source: J Exp Clin Cancer Res. 2018 Nov 9;37:272. doi: 10.1186/s13046-018-0935-8 (PMC6230241; doi:10.1186/s13046-018-0935-8)
Supplement: Supplementary file 7 — Figure S6. Representative FCM graphs of SGC7901 (a) and SGC7901/ADR (b) corresponding to the data in Fig. 5d. The treatments were the same as those in Fig. 5d. (PPTX 368 kb) [file 13046_2018_935_MOESM7_ESM.pptx]

## Slide 1
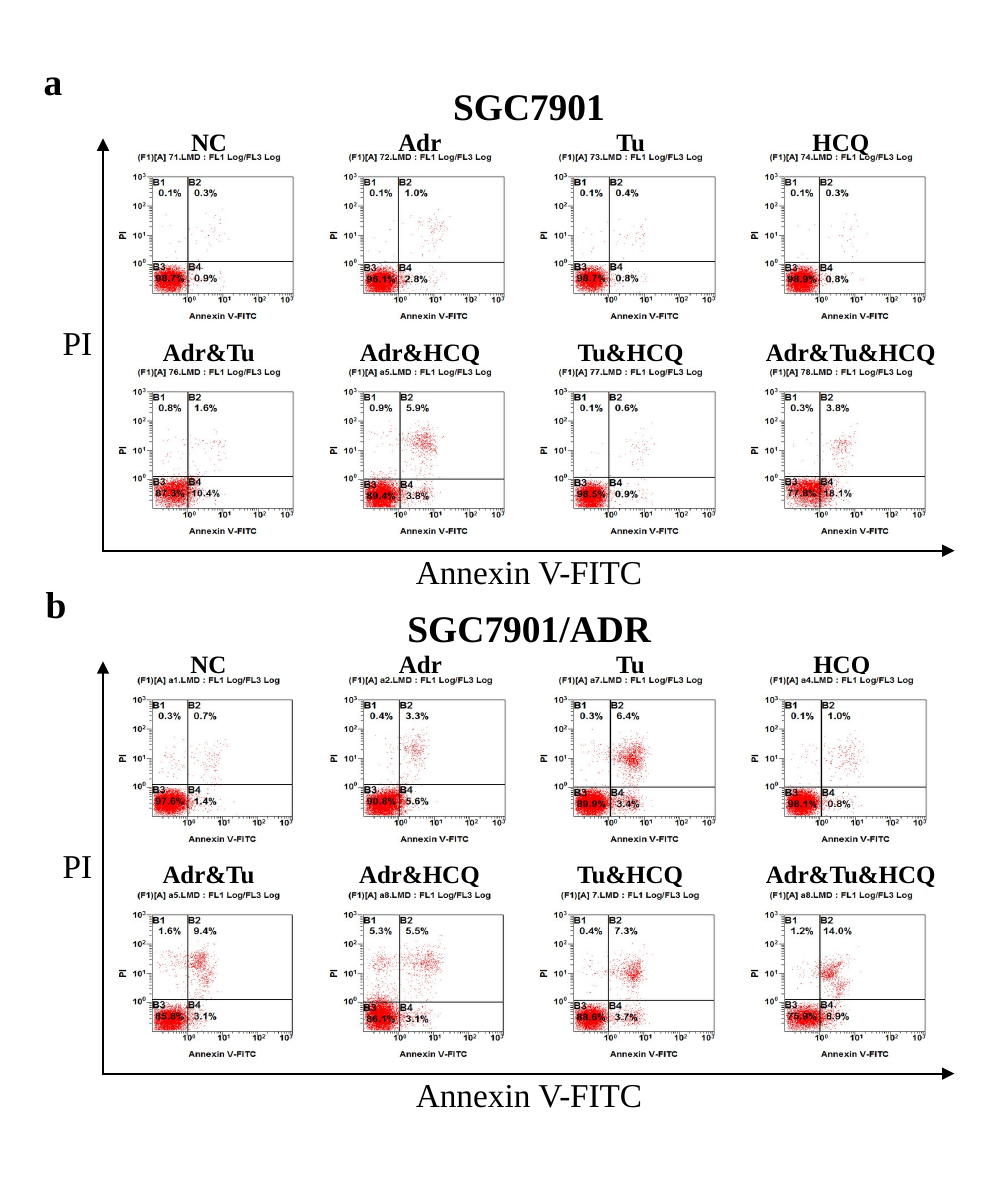

a
SGC7901
NC
Adr
Tu
HCQ
PI
Adr&Tu
Adr&HCQ
Tu&HCQ
Adr&Tu&HCQ
Annexin V-FITC
b
SGC7901/ADR
NC
Adr
Tu
HCQ
PI
Adr&Tu
Adr&HCQ
Tu&HCQ
Adr&Tu&HCQ
Annexin V-FITC
